# Surrogate Markers of Visceral Adiposity in Young Adults: Waist Circumference and Body Mass Index Are More Accurate than Waist Hip Ratio, Model of Adipose Distribution and Visceral Adiposity Index

**DOI:** 10.1371/journal.pone.0114112

**Published:** 2014-12-05

**Authors:** Susana Borruel, José F. Moltó, Macarena Alpañés, Elena Fernández-Durán, Francisco Álvarez-Blasco, Manuel Luque-Ramírez, Héctor F. Escobar-Morreale

**Affiliations:** 1 Department of Endocrinology & Nutrition, Universidad de Alcalá & Hospital Universitario Ramón y Cajal & Instituto Ramón y Cajal de Investigación Sanitaria IRYCIS & Centro de Investigación Biomédica en Red Diabetes y Enfermedades Metabólicas Asociadas (CIBERDEM), Madrid, Spain; 2 Department of Radiology, Hospital Universitario 12 de Octubre, Madrid, Spain; University of East Anglia, United Kingdom

## Abstract

Surrogate indexes of visceral adiposity, a major risk factor for metabolic and cardiovascular disorders, are routinely used in clinical practice because objective measurements of visceral adiposity are expensive, may involve exposure to radiation, and their availability is limited. We compared several surrogate indexes of visceral adiposity with ultrasound assessment of subcutaneous and visceral adipose tissue depots in 99 young Caucasian adults, including 20 women without androgen excess, 53 women with polycystic ovary syndrome, and 26 men. Obesity was present in 7, 21, and 7 subjects, respectively. We obtained body mass index (BMI), waist circumference (WC), waist-hip ratio (WHR), model of adipose distribution (MOAD), visceral adiposity index (VAI), and ultrasound measurements of subcutaneous and visceral adipose tissue depots and hepatic steatosis. WC and BMI showed the strongest correlations with ultrasound measurements of visceral adiposity. Only WHR correlated with sex hormones. Linear stepwise regression models including VAI were only slightly stronger than models including BMI or WC in explaining the variability in the insulin sensitivity index (yet BMI and WC had higher individual standardized coefficients of regression), and these models were superior to those including WHR and MOAD. WC showed 0.94 (95% confidence interval 0.88–0.99) and BMI showed 0.91 (0.85–0.98) probability of identifying the presence of hepatic steatosis according to receiver operating characteristic curve analysis. In conclusion, WC and BMI not only the simplest to obtain, but are also the most accurate surrogate markers of visceral adiposity in young adults, and are good indicators of insulin resistance and powerful predictors of the presence of hepatic steatosis.

## Introduction

Visceral adiposity is associated with cardiovascular and metabolic disorders [1] including insulin resistance [2], low-grade chronic inflammation [3], type 2 diabetes [4], dyslipidemia [5], polycystic ovary syndrome (PCOS) [6], male hypogonadism [7], nonalcoholic fatty liver disease [8], hypertension [9] and several cancers [10]. Amelioration of visceral adiposity is of importance for the prevention and management of most of these disorders [11].

Assessment of visceral adiposity is advised in many situations and is becoming routine clinical practice nowadays. However, the most accurate measurements of visceral adiposity – magnetic resonance imaging, computed tomography and ultrasound – are seldom available for most clinicians, since the imaging techniques involved are expensive, may involve exposure to radiation, or require intensive training [12]. Hence, imaging techniques are frequently substituted by surrogate indexes of visceral adiposity.

Waist circumference (WC) and waist-hip ratio (WHR) are the most commonly used surrogate indexes of visceral adiposity. In fact, increased WC is a requisite for the diagnosis of the metabolic syndrome according to the International Diabetes Federation [13].

Visceral adiposity index (VAI) has been proposed as and indicator of visceral adipose function and insulin sensitivity that may reflect cardiometabolic risk [14]. This index derived from the study of 315 Italian subjects aged 19–83 yr-old and presenting with body mass index (BMI) values between 20 and 30 kg/m^2^, not having evidence for diabetes mellitus or impaired fasting glucose, high blood pressure, dyslipidemia, metabolic syndrome and cardiovascular disease [14]. VAI included a sex-specific model of adipose distribution (MOAD) based on the linear relationship between WC and body mass index (BMI) in each sex that is corrected for fat function introducing triglycerides and high-density lipoprotein (HDL)-cholesterol concentrations into the equation [14]. MOAD showed statistically significant correlations with the area and volume of visceral adipose tissue assessed by magnetic resonance imaging is a subset of 26 metabolically healthy individuals [14]. In a subsequent series of 1,498 primary-care patients, the number of patients with metabolic syndrome, diabetes, high blood pressure, dyslipidemia, coronary heart disease and cerebrovascular disease increased significantly across VAI quintiles in parallel with the increase in age [14]. Moreover, in a mixed series of 74 patients with type 1 diabetes, type 2 diabetes, nonalcoholic fatty liver disease and PCOS, VAI correlated negatively with glucose disposal rate M-values during a euglycemic hyperinsulinemic clamp, whereas WC and BMI did not [14].

However, the application of the VAI in different populations and in clinical series of patients with metabolic disorders such as nonalcoholic fatty liver disease, obstructive sleep apnea and PCOS yielded conflicting results regarding its role as marker of abdominal adiposity, insulin resistance and risk of disease [15]–[22].

We aimed to evaluate WC, WHR, MOAD and VAI as surrogate indexes of visceral adiposity, objectively assessed by ultrasound examination in young adults including healthy women, women with PCOS, and healthy men, presenting with or without obesity.

## Subjects and Methods

### 1. Subjects

This study derived from a previous work aiming to assess global and visceral adiposity in women with PCOS [23]. Ninety-nine young Caucasian adults, including 20 women without androgen excess, 53 women with PCOS, and 26 men were included in the present analysis of the data. Seven control women, 21 PCOS patients and 7 men presented with obesity as defined by a body mass index (BMI) ≧30 kg/m^2^ [24]. We included patients with PCOS in addition to non-obese and obese healthy women and men to include a subset of patients with global and abdominal adiposity and insulin resistance, because PCOS associates these disorders irrespective of obesity [23].

PCOS was defined by the presence of ovulatory dysfunction together with clinical hyperandrogenism and/or hyperandrogenemia, after exclusion of specific etiologies [25]. All the patients suffered the classic hyperandrogenic PCOS phenotype and, even when ovarian morphology was not analyzed, by having hyperandrogenism and oligoovulation all patients also fulfilled all the current definitions of PCOS [25]–[27]. On the contrary, we considered as controls, women presenting without menstrual and ovulatory dysfunction and who had no evidence of androgen excess. The methods and assays used to diagnose in the patients and to exclude hyperandrogenic disorders in healthy women have been described in detail elsewhere [23], [28], [29] and, besides extensive hormonal testing, included a standard 2-h 75 g oral glucose tolerance test that permitted the calculation of the composite insulin sensitivity index from glucose and insulin concentrations [30]. Total body fat mass was estimated using a body fat monitor (Omron BF 300, Omron Corp., Kyoto, Japan) and was expressed as percentage of total body mass.

None of the subjects had received treatment with any drug known to interfere with sex hormone secretion and metabolism such as oral contraceptives, antiandrogens or insulin sensitizers for the previous 6 months. Written informed consent was obtained from all the adult participants. Minors gave verbal consent that was confirmed in writing and in their behalf by their legal guardians. The study and the informed consent procedures were approved by the Ethics Committee of Hospital Universitario Ramón y Cajal.

### 2. Surrogate indexes of visceral adiposity

We used a non-stretchable measuring tape to measure waist and hip circumferences. The smallest abdominal circumference between the lowest rib and the iliac crest was used as WC. WHR was calculated by dividing WC by the hip circumference at the level of greater trochanters. MOAD was calculated according to the following formulae:
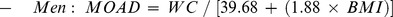


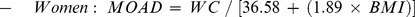
and VAI was then calculated as:
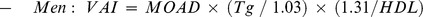


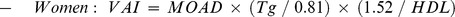
where WC was introduced in cm and BMI was introduced in kg/m^2^, Tg were triglycerides levels in mmol/l, and HDL were high-density lipoprotein cholesterol concentrations in mmol/l.

### 3. Ultrasound assessment of adipose tissue depots

In experienced hands, ultrasonography is a precise and reliable method for evaluation of visceral fat, showing an excellent correlation and concordance with CT scan [31]–[33]. Adipose tissue depots were estimated using a Toshiba Nemio XG SSA-580A Diagnostic Ultrasound System (Toshiba Medical Systems S.A., Alcobendas, Madrid, Spain) by an experienced sonographer who was blinded for the PCOS status of the women studied here.

Subjects were examined in the fasting state and in the supine position, and were asked to hold their breath during the examination while the frozen images were taken, to avoid the influence of the respiratory status or abdominal wall tension. Special care was taken to keep the probe just touching the skin to prevent compression of the fat layers.

Minimum and maximum subcutaneous, preperitoneal, intraperitoneal, mesenteric, epicardial and perirenal adipose tissue thicknesses were measured as described [23]. Three measures of intraperitoneal fat thickness were obtained: distance from the fascia of rectus abdominis muscle to vertebral column, distance from peritoneum to vertebral column, and distance from linea alba to vertebral column. Mesenteric fat thickness was measured as described by Liu *et al.* [34] and perirenal fat thickness was estimated as the distance from the perirenal fascia to the renal surface on a long-axis view of the right kidney. Finally, epicardial fat thickness was measured in the free wall of the right ventricle from still images obtained by two-dimensional transthoracic echocardiography using a 3 MHz transducer, as described by Ahn *et al.* [35]. Mediastinal fat presenting as an echo-lucent area above the parietal pericardium was excluded from the measurement. Values obtained in long-axis and short axis view were similar and, hence, only values obtained from long-axis views were submitted to statistical analysis. For each ultrasound measurement of fat thickness, intra-operator coefficients of variation (CVs) were calculated by repeating 20 measurements in a single individual. Four cycles of five consecutive measurements, separated at 10 minutes intervals, were used to calculate CVs. The CVs were 4.5% for the distances from rectus abdominis and linea alba to vertebral column, 4.9% for the distance between peritoneum to vertebral column, 5.8% for preperitoneal visceral fat, 6.6% for minimum and 5.9% for maximum subcutaneous adipose tissue thickness, 9.8% for perirrenal fat, and 15.5% mesenteric fat (15%), which was the only measurement of fat thickness showing a coefficient of variation above 10%.

### 4. Ultrasound assessment of hepatic steatosis

Hepatic steatosis was estimated using a 3.5-MHz transducer as described by Saadeh *et al.* [36] Hepatic steatosis was diagnosed when the echogenicity of the liver was higher than the echogenicity of the right kidney, and graded as follows: grade 0, normal echogenicity; grade 1, slight, diffuse increase in fine echoes in liver parenchyma with normal visualization of diaphragm and intrahepatic vessel borders; grade 2, moderate, diffuse increase in fine echoes with slightly impaired visualization of intrahepatic vessels and diaphragm; grade 3, marked increase in fine echoes with poor or nonvisualization of the intrahepatic vessel borders, diaphragm, and posterior right lobe of the liver.

### 5. Statistical analysis

Nominal and ordinal variables were analyzed by Pearson's χ^2^ test. Continuous variables are reported as means ± SD (range) in the text or as means ± SEM in the figures. The Kolmogorov-Smirnov statistic was applied to continuous variables. We applied logarithmic or square-root transformations as needed to ensure normal distribution of the variables. Relationships between the surrogate indexes of visceral adiposity with the thickness of the different adipose tissue depots and other clinical and biochemical variables were analyzed by Pearson's correlation analysis.

Differences in phenotypic variables among men, women and patients with PCOS were analyzed by univariate one-way general linear models (GLMs). Surrogate markers of visceral adiposity were tested by univariate two-way GLMs in which group of subjects and presence or absence of obesity were introduced as independent variables. A two-way GLM is a statistical model where changes in a continuous and normally distributed dependent variable are explained by a linear combination of functions of several independent explanatory variables (group of subjects and obesity in our study). In essence, a two-way GLM is similar to two-way analysis of variance, although the computational background of both tests is entirely different. The two-way GLM would test if there are differences according to one or both dependent variables, and/or if there is an interaction indicating that the effect of one of the independent variables on the dependent variable is not the same at all levels of the other independent variable (i.e., that the effect of obesity on a dependent variable is not the same in men, control women and patients with PCOS). Given that obesity was included as one of the dependent variables, and that the groups of subjects were not different in terms of BMI, the impact of both obesity and BMI was automatically considered by the GLM when analyzing differences between men, control women and patients with PCOS. Hence, BMI was not introduced as dependent variable in these comparisons. Also, in these GLMs age was introduced as a covariate to control for a difference in age among patients with PCOS and the other groups.

One-way GLMs were used to explore differences in surrogate indexes of visceral adiposity and BMI in subjects depending on the grade of hepatic steatosis. Only when univariate GLMs showed differences among the group of subjects, pairwise comparisons among the three groups of individuals were analyzed by the least significant difference test for *post-hoc* comparisons. Correlation and multiple linear regression analyses were conducted as described below. Receiver operating characteristic curve (ROC) analysis was used to assess the accuracy of surrogate indexes of visceral adiposity as diagnostic predictors of the presence or absence of hepatic steatosis. We used SPSS Statistics 17.0 (SPSS Ibérica, Madrid, Spain) and set α = 0.05 as the level of statistical significance for all the analyses.

## Results

### 1. Clinical, hormonal and metabolic variables in healthy women, patients with PCOS and men

Obesity was present in 7 of the 20 control women, 21 of the 53 women with PCOS, and in 7 of the 26 men (χ^2^ = 1.232, *P* = 0.540). No differences among them were found for BMI [control women: 26±7 (17–38) kg/m^2^; PCOS patients: 30±9 (19–52) kg/m^2^; men: 30±9 (22–56) kg/m^2^, *P* = 0.214]. Patients with PCOS were younger compared with control women and men [control women: 30±5 (20–38) yr; PCOS patients: 25±6 (14–39) yr; men: 33±5 (24–41) yr, *P*<0.001]. Therefore, age was introduced as a covariate in the comparisons of all other variables.

The clinical, metabolic and hormonal characteristics of the three groups of subjects compared in the study are summarized in Table 1. When expressed as percentage of total body mass, the higher fat mass was observed in women with PCOS followed by control women and finally men.

**Table 1:** Clinical, metabolic and hormonal variables in control women, patients with polycystic ovary syndrome (PCOS) and men.

	Control women			PCOS patients			Men			*P*
	(n = 25)			(n = 55)			(n = 26)			
Systolic blood pressure (mmHg)*	110	±	9	114	±	14	130	±	15	<0.001
Diastolic blood pressure (mmHg)†	72	±	8	74	±	10	80	±	10	0.003
Total body fat mass (%)* ^,^ ‡	29	±	11	33	±	9	25	±	9	<0.001
Hirsutism score‡	1.8	±	1.4	9.8	±	5.9	NA			<0.001
Total testosterone (nmol/l)* ^,^ ‡	1.7	±	0.5	2.2	±	0.9	17.6	±	6.2	<0.001
Free testosterone (pmol/l)* ^,^ ‡	24	±	10	38	±	21	413	±	111	<0.001
SHBG (nmol/l)*	49	±	18	41	±	23	27	±	11	<0.001
DHEAS (µmol/l)	6.2	±	3.4	6.8	±	3.1	6.3	±	2.2	0.652
Androstenedione (nmol/l)‡ ^,^ §	9.4	±	3.5	12.9	±	4.5	9.1	±	3.1	0.007
Estradiol (pmol/l)* ^,^ ‡	305	±	169	169	±	77	117	±	37	<0.001
Luteinizing hormone (mU/mL)*	5.2	±	1.8	7.3	±	7.1	3.7	±	1.7	0.003
FSH (mU/ml)*	5.7	±	2.1	5.0	±	1.1	3.2	±	1.8	<0.001
Fasting glucose (mmol/l)	4.9	±	0.6	5.1	±	0.7	5.3	±	0.5	0.097
Fasting insulin (pmol/l)† ^,^ ‡	26	±	17	67	±	76	51	±	40	0.017
Insulin sensitivity index† ^,^ ‡	10.0	±	4.0	7.3	±	5.1	6.9	±	4.7	0.017
Leukocytes (cells/µl)	6290	±	1350	6853	±	1971	6023	±	1426	0.495
Ferritin (pmol/l)* ^,^ ‡	92	±	101	135	±	101	369	±	198	<0.001
Total cholesterol (mmol/l)	4.6	±	0.9	4.8	±	1.3	4.9	±	0.9	0.149
LDL-cholesterol (mmol/l)	2.7	±	0.6	3.0	±	1.1	3.2	±	0.8	0.064
HDL-cholesterol (mmol/l)† ^,^ ‡	1.6	±	0.5	1.3	±	0.3	1.2	±	0.3	0.005
Triglycerides (mmol/l)† ^,^ ‡	0.8	±	0.3	0.9	±	0.4	1.2	±	0.6	0.019
Aspartate aminotransferase (U/L)*	19	±	7	17	±	5	23	±	9	0.002
Alanine aminotransferase (U/L)*	19	±	10	21	±	12	31	±	17	0.013
γ-Glutamyltransferase (U/L)	21	±	13	24	±	22	30	±	17	0.104
Alkaline phosphatase (U/L)	61	±	22	70	±	18	66	±	19	0.311

*Abbreviations* DHEAS, dehydroepiandrosterone-sulfate; HDL, high-density lipoprotein; LDL, low-density lipoprotein; NA, not applicable; SHBG, sex hormone-binding globulin.

Data are means ± SD. Data were submitted to univariate general linear models followed by the least significant difference *post hoc* test. Age was introduced as a covariate in the comparisons because patients with PCOS were younger compared with control women and men.

* *p*<0.05 or less for the difference between men and both control women and patients with PCOS.

† *p*<0.05 or less for the difference between men and control women.

‡ *p*<0.05 or less for the difference between patients with PCOS and control women.

§ *p*<0.05 or less for the difference between men and women with PCOS.

Systolic blood pressure was higher in men compared with both groups of women, whereas diastolic blood pressure was increased in men only when compared with control women. The hirsutism score was increased in patients with PCOS compared with control women. Total and free testosterone concentrations were higher in men compared with control women with patients with PCOS presenting with intermediate values that were increased compared with control women. Opposite changes were found for serum estradiol concentrations. Sex hormone-binding globulin concentrations were reduced in men compared with both groups of women. Dehydroepiandrosterone-sulfate concentrations were similar in all the groups but androstenedione levels were increased in patients with PCOS compared with both control women and men.

Regarding metabolic and inflammatory variables, fasting glucose concentrations were similar in the three groups of subjects, whereas fasting insulin levels were increased, and the composite insulin sensitivity index derived from an oral glucose tolerance test was decreased, in both men and patients with PCOS compared with control women. In patients with PCOS and men, HDL-cholesterol concentrations were lower and serum triglycerides were higher than those of control women. Men presented increased transaminases compared with both groups of women, yet no differences were observed for γ-glutamyltransferase and alkaline phosphatase.

### 2. Surrogate indexes of visceral adipose tissue in healthy women, patients with PCOS and men as a function of obesity

**Figure 1:**
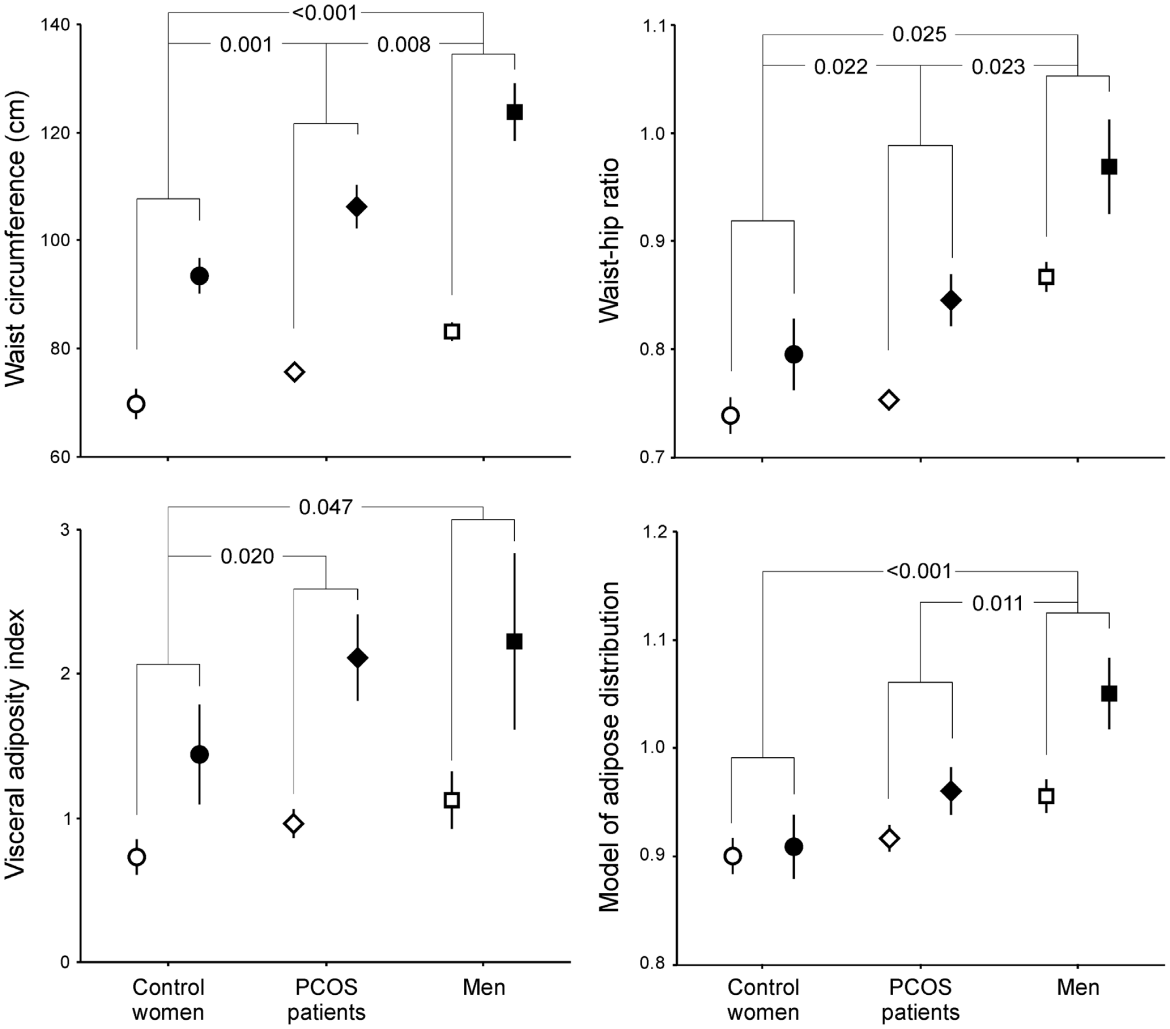
Surrogate indexes of visceral adiposity in control women (circles, n = 20), women with polycystic ovary syndrome (PCOS, diamonds, n = 53) and men (squares, n = 26) as a function of obesity (defined as BMI ≧30 kg/m^2^, white symbols  =  non-obese subjects, black symbols  =  obese individuals). Data are means ± SEM and were submitted to univariate general linear models introducing groups of subjects and obesity as independent variables and age as a covariate to correct the results for a difference in age among patients with PCOS with the other groups. Obese subjects showed increased total body fat mass values when compared with non-obese individuals (*P*<0.001 for all indexes with the exception of MOAD, *P* = 0.011), irrespective of sex and PCOS. *P* values indicate the differences between control women, women with PCOS and men, irrespective of the presence or absence of obesity. No interaction was found between obesity and groups of subjects in any of the measurements of the indexes of visceral adiposity.

The differences in surrogate indexes of visceral adiposity between healthy women, patients with PCOS and men, and between non-obese and obese subjects, were larger for waist circumference, followed by WHR, VAI and MOAD (Figure 1). WC was the index showing the smallest variation in all the groups of subjects studied here (Figure 1).

All indexes were higher in obese persons compared to non-obese subjects, irrespective of being healthy women, patients with PCOS or men (Figure 1). Regarding the comparison between groups, the largest WC and WHR were observed in men and the smallest were observed in control women, with patients with PCOS showing intermediate values that were smaller than those for men and larger than those of control women (Figure 1). Similar findings were observed for VAI and MOAD, yet VAI failed to reveal any difference between men and patients with PCOS, and MOAD did not detect any difference between patients with PCOS and control women (Figure 1).

### 3. Correlation of surrogate indexes of visceral adiposity with ultrasound assessment of adipose tissue depots

Of the indexes of visceral adiposity studied here, WC and BMI showed the strongest correlations with all measurements of visceral fat thickness. Furthermore, WC and BMI showed also strong correlations with measurements of subcutaneous and preperitoneal fat thickness (Table 2). Of note, with the exception of the correlation with epicardial fat thickness, all the correlations of WC and BMI with subcutaneous and visceral fat thicknesses measured here showed coefficients of correlation well above 0.5 (Table 2). When considering the strength of the correlations with ultrasound measurements, WHR took the third place after WC and BMI, and was followed by VAI and MOAD (Table 2).

**Table 2:** Correlation of the thickness of adipose tissue depots as assessed by ultrasound with surrogate indexes of visceral adiposity and with body mass index when considering control women, patients with PCOS and men as a whole.

	Subcutaneous		Preperitoneal	Intraperitoneal			Mesenteric	Perirenal	Epicardial
	Minimum	Maximum		RA-VC	P-VC	LA-VC			
Waist circumference	0.803*	0.836	0.639*	0.793*	0.761*	0.796*	0.808*	0.662*	0.464*
Waist-hip ratio	0.461*	0.521*	0.495*	0.703*	0.701*	0.707*	0.610*	0.607*	0.287†
Visceral adiposity index	0.510*	0.561*	0.504*	0.582*	0.557*	0.596*	0.605*	0.431*	0.281†
Model of adipose distribution	0.434*	0.469*	0.432*	0.523*	0.501*	0.524*	0.471*	0.427*	0.256†
Body mass index	0.838*	0.861*	0.601*	0.738*	0.694*	0.735*	0.789*	0.614*	0.478*

Values are Pearson's correlation coefficients. Logarithmic or square root transformations were applied to raw data as needed to ensure a normal distribution of the variables.

*P<0.001;

† *P*<0.01.

*Abbreviations* LA-VC, Linea alba – vertebral column; P-VC, Peritoneum – vertebral column; RA-VC; Rectus abdominis – vertebral column.

### 4. Correlation of surrogate indexes of visceral adiposity with clinical and biochemical variables related to the metabolic syndrome, insulin resistance, low-grade chronic inflammation, hepatic steatosis and sexual function

Focusing on strong correlations (i.e. coefficient of correlation>0.5), WC correlated directly with both systolic and diastolic blood pressure, fasting insulin, leukocytes count, alanine aminotransferase and γ-glutamyltransferase, and negatively with the composite insulin sensitivity index (Table 3). BMI correlated directly with diastolic blood pressure, fasting insulin and leukocyte count, WHR correlated directly with free testosterone, alanine aminotransferase and γ-glutamyltransferase, VAI correlated directly with fasting insulin and negatively with the insulin sensitivity index, whereas MOAD only correlated directly with γ-glutamyltransferase (Table 3). There were many weaker correlations (namely, coefficients of correlation <0.5, Table 3). Of note, WHR and MOAD were the surrogate indexes of visceral adiposity that correlated better with sexual steroids and gonadotropins concentrations (Table 3).

**Table 3:** Correlation of the surrogate markers of abdominal adiposity and body mass index with clinical, hormonal and metabolic variables when considering control women, patients with PCOS and men as a whole.

	Waist circumference	Waist-hip ratio	Visceral adiposity index	Model of adipose distribution	Body mass index
Systolic blood pressure (mmHg)	0.549*	0.468*	0.387*	0.382*	0.483*
Diastolic blood pressure (mmHg)	0.651*	0.459*	0.412*	0.387*	0.632*
Total testosterone (nmol/l)	0.127	0.466*	0.031	0.290†	−0.051
Free testosterone (pmol/l)	0.179	0.520*	0.065	0.315†	−0.002
Sex hormone-binding globulin (mmol/l)	−0.478*	−0.485*	−0.405*	−0.360*	−0.406*
Androstenedione (nmol/l)	−0.046	0.003	0.108	0.085	−0.088
Dehydroepiandrosterone-sulfate (µmol/l)	−0.099	−0.078	−0.039	−0.012	−0.119
Estradiol (pmol/l)	−0.157	−0.276†	−0.136	−0.201‡	−0.064
Luteinizing hormone (mU/mL)	−0.224‡	−0.230‡	−0.136	−0.101	−0.207‡
Follicle-stimulating hormone (mU/ml)	−0.277†	−0.423*	−0.165	−0.324†	−0.150
Fasting glucose (mmol/ll)	0.357*	0.352†	0.220‡	0.270‡	0.321*
Fasting insulin (pmol/l)	0.548*	0.430*	0.556*	0.386*	0.516*
Insulin sensitivity index	−0.501*	−0.433*	−0.532*	−0.372*	−0.454*
Leukocytes (cells/µl)	0.541*	0.238‡	0.495*	0.255‡	0.587*
Ferritin (pmol/l)	0.314†	0.459*	0.129	0.342†	0.196
Aspartate aminotransferase (U/L)	0.250‡	0.289‡	0.091	0.233‡	0.178
Alanine aminotransferase (U/L)	0.550*	0.567*	0.390*	0.487*	0.437*
γ-Glutamyltransferase (U/L)	0.607*	0.597*	0.445*	0.530*	0.489*
Alkaline phosphatase (U/L)	0.377*	0.204‡	0.295†	0.227‡	0.375*

Values are Pearson's correlation coefficients. Logarithmic or square root transformations were applied to raw data as needed to ensure a normal distribution of the variables.

*P<0.001;

† *P*<0.01;

‡ *P*<0.05.

Regarding the possible roles of these indexes as markers of insulin sensitivity, the correlations of VAI with fasting insulin and the insulin sensitivity index were slightly better than those of WC and BMI, and were definitely stronger than those of WHR and MOAD (Table 3). Next, we included each surrogate index of visceral adipose tissue and BMI in separate multiple stepwise regression models that also included the insulin sensitivity index as dependent variable and free testosterone, estradiol, age, ferritin and leukocytes counts as independent variables (because age, sex hormones, iron stores and inflammation may influence insulin sensitivity).

As predictors of the insulin sensitivity index, the first model (adjusted R^2^ = 0.261, *F* = 17.6, *P*<0.001) retained WC (β = −0.40) and leukocyte count (β = −0.19); the second model (adjusted R^2^ = 0.257, *F* = 17.3, *P*<0.001) retained WHR (β = −0.34) and leukocyte count (β = −0.33); the third model (adjusted R^2^ = 0.275, *F* = 12.9, *P*<0.001) retained VAI (β = −0.36), leukocyte count (β = −0.25) and ferritin (β = −0.16); the fourth model (adjusted R^2^ = 0.209, *F* = 13.4, *P*<0.001) retained MOAD (β = −0.26) and leukocyte count (β = −0.34) and the fifth model (adjusted R^2^ = 0.218, *F* = 27.2, *P*<0.001) retained only the BMI (β = −0.48). Therefore, the highest β coefficients were those of BMI and WC, followed by VAI, WHR and MOAD.

### 5. Surrogate markers of visceral adiposity and ultrasound assessment of hepatic steatosis

We observed no differences in the frequency of hepatic steatosis among control women, patients with PCOS and men (20%, 36% and 40% respectively; χ^2^ = 2.609; *P* = 0.271), whereas steatosis was eight times more prevalent in obese subjects compared with non-obese individuals (80% vs. 9% respectively; χ^2^ = 50.050; *P*<0.001).

**Figure 2:**
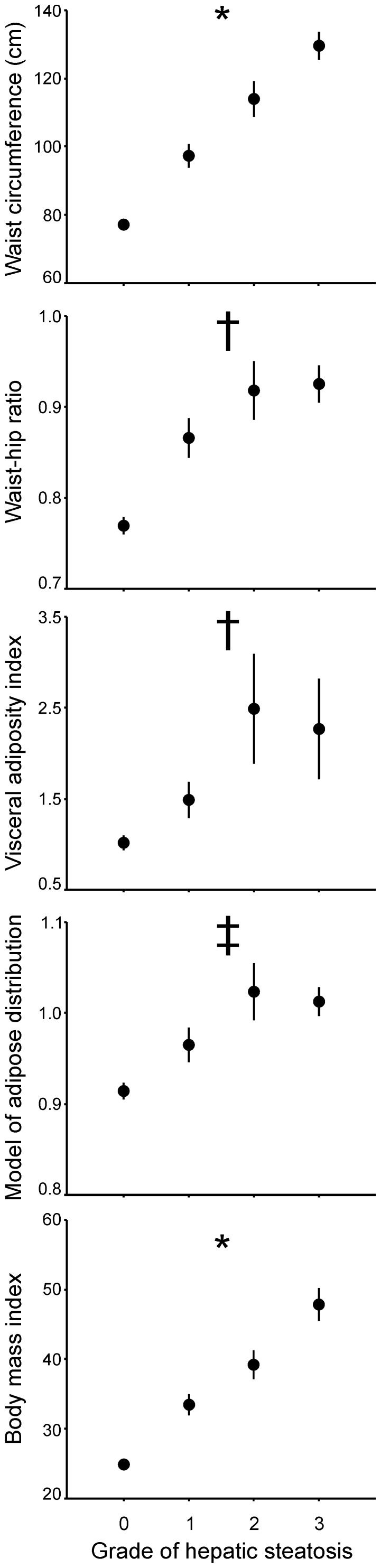
Surrogate indexes of visceral adiposity and BMI depending on the grade of hapatic steatosis as estimated by ultrasound in the 99 subjects included in the study, irrespective of sex, PCOS and obesity. Hepatic steatosis was graded as follows: grade 0, normal echogenicity; grade 1, slight, diffuse increase in fine echoes in liver parenchyma with normal visualization of diaphragm and intrahepatic vessel borders; grade 2, moderate, diffuse increase in fine echoes with slightly impaired visualization of intrahepatic vessels and diaphragm; grade 3, marked increase in fine echoes with poor or nonvisualization of the intrahepatic vessel borders, diaphragm, and posterior right lobe of the liver. Data are means ± SEM and were submitted univariate general linear models introducing grade of hepatic steatosis as independent variable and age as a covariate. * All comparisons between grades of steatosis showed statistically significant differences with *P*<0.05 or less. † Values in grades 1, 2 and 3 were similar and higher than those observed in grade 0. ‡ Values in grades 1, 2 and 3 steatosis were higher than those observed in grade 0, and grade 2 steatosis also showed higher values compared with grade 1.

**Figure 3:**
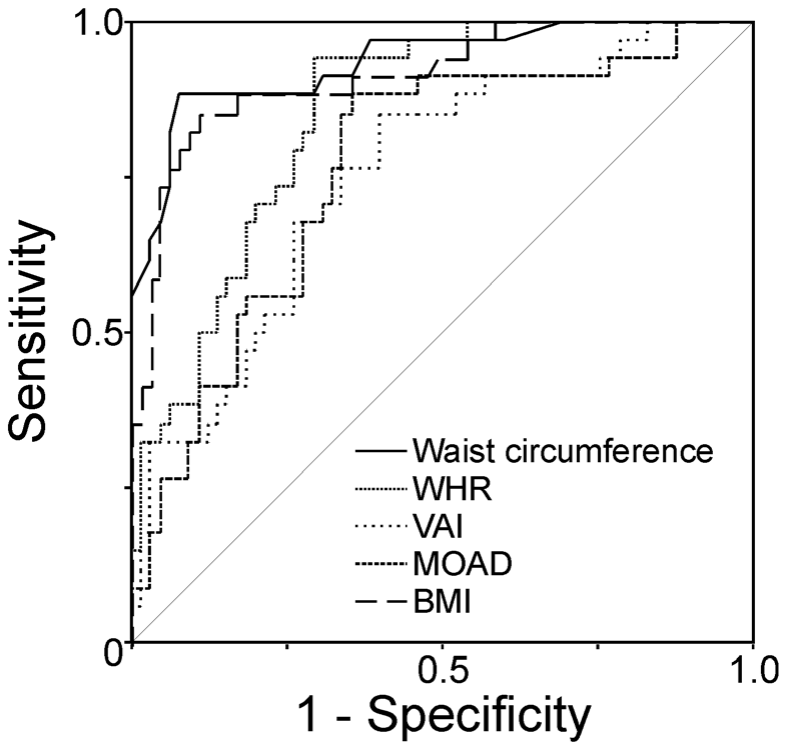
Receiver operating characteristic (ROC) curve analysis of the diagnostic performance of surrogate indexes of visceral adiposity and BMI for the presence or absence of hepatic steatosis. All ROC curves were statistically significant with *P*<0.001. The best area under de ROC curve was that of waist circumference (0.935±SE 0.027) which was better than those of WHR (difference 0.08, 95% confidence interval 0.01–0.16), VAI (difference 0.18, 95% confidence interval 0.10–0.26 and MOAD (difference 0.17, 95% confidence interval 0.08–0.26), but was not different than that of BMI (difference 0.02, 95% confidence interval −0.01 – to 0.05, *P* = 0.155). Similarly, the area under the ROC curve of BMI was better compared with those of VAI (difference 0.16, 95% confidence interval 0.08–0.24, *P*<0.001) and MOAD (difference 0.15, 95% confidence interval 0.04–0.26, *P* = 0.007) but was not different than that of WHR (difference 0.06, 95% confidence interval 0.03 – to 0.15, *P* = 0.176).

There was a graded increase in WC and BMI between the groups defined by the severity of hepatic steatosis (Figure 2). Such an increase was not clearly observed for other surrogate indexes of visceral adiposity, which were less sensitive in detecting differences among the subgroups of patients with different grades of hepatic steatosis (Figure 3).

We then assessed the possible diagnostic accuracy of surrogate indexes of visceral adiposity for the presence or absence of hepatic steatosis, considering grades 1, 2 and 3 as a whole, using ROC curve analysis (Figure 3). The areas under the ROC curve ± SE were 0.935±0.027 for waist circumference, 0.853±0.037 for WHR, 0.754±0.05 for VAI, 0.764±0.05 for MOAD and 0.914±0.031 for BMI. The area under the ROC curve of WC was better compared with those of WHR (difference 0.08, 95% confidence interval 0.01–0.16, *P* = 0.036), VAI (difference 0.18, 95% confidence interval 0.10–0.26, *P*<0.001) and MOAD (difference 0.17, 95% confidence interval 0.08–0.26, *P*<0.001) but was not different than that of BMI (difference 0.02, 95% confidence interval −0.01 – to 0.05, *P* = 0.155). Similarly, area under the ROC curve of BMI was better compared with those of VAI (difference 0.16, 95% confidence interval 0.08–0.24, *P*<0.001) and MOAD (difference 0.15, 95% confidence interval 0.04–0.26, *P* = 0.007) but was not different than that of WHR (difference 0.06, 95% confidence interval 0.03 – to 0.15, *P* = 0.176).

These results indicate that WC had a 93.5% chance of predicting the presence of hepatic steatosis in our mixed population of young adults of both sexes, and a cut-off value of WC above or equal to 91.5 cm had 88% sensitivity and 92% specificity for the presence of hepatic steatosis in them. Similarly, BMI had a 91.4% predicting the presence of hepatic steatosis, and a cut-off value of BMI above or equal to 29.7 kg/m^2^ had 85% sensitivity and 89% specificity for the presence of hepatic steatosis.

## Discussion

Our present results indicate that, in young adults, WC and BMI are the easiest to obtain and also the most accurate markers of visceral adiposity, as assessed by ultrasound, of the surrogate markers studied here. All the coefficients of correlation between WC, BMI and the thicknesses of abdominal adiposity were well-above 0.5. Such strong correlations were observed not only with measurements of intraperitoneal adipose depots such as the distances between rectus abdominis, peritoneum or linea alba to the vertebral column, mesenteric and perirenal fat, but also with objective measurements of preperitoneal and subcutaneous abdominal fat. Only the coefficient of correlation of WC and BMI with epicardial fat was slightly below 0.5. Compared with waist circumference, WHR, VAI and MOAD showed weaker although statistically significant correlations with objective ultrasound measurements of the thickness of visceral adipose tissue depots.

This is possibly related to the fact that, being the simplest indexes to obtain, WC and BMI showed the smallest variation among the surrogate indexes of visceral adiposity studied here in all the groups of subjects included in the study, as clearly shown in Figure 2. Also, as depicted in Figure 1, only WC and WHR, and not VAI or MOAD, were able to detect all the differences between control women, patients with PCOS and men in the thickness of intraperitoneal fat detected by ultrasound [23], both in the non-obese and obese subgroups.

Because WC measures both subcutaneous and visceral abdominal fat, the rationale behind the development of VAI was to find an easy to obtain clinical marker of visceral adiposity that improved WC by adding variables addressing global adiposity such as BMI and variables addressing visceral adipose tissue function such as serum triglycerides and HDL-cholesterol concentrations [14]. One of the theoretical advantages of VAI over WC would be that VAI should give a better insight about visceral adipose function and insulin sensitivity, and its increase would be strongly associated with cardiometabolic risk [14]. The correlations between surrogate markers of visceral adiposity with clinical variables related to the metabolic and cardiovascular complications of abdominal obesity in our series do not support this hypothesis.

Compared with VAI, WC and BMI showed similarly strong correlations with fasting insulin and insulin resistance estimated by the decrease in the composite insulin sensitivity index, but also showed several strong correlations with systolic and diastolic blood pressure values and with serum transaminases that were considerably weaker (r<0.5) in the case of VAI, MOAD and WHR.

Moreover, WC and BMI showed excellent diagnostic performances for the detection of hepatic steatosis. In fact, the area under the ROC curve of WC as a marker of the presence of absence of hepatic steatosis was larger than those of VAI, WHR and MOAD, and that of BMI was larger than those of VAI and MOAD. Moreover, both WC and BMI showed a graded increase as a function of the grade of hepatic steatosis as assessed by ultrasound, whereas other surrogate indexes of visceral adiposity were only different between subjects with or without steatosis, and not between subjects with different grades of steatosis. To this regards, VAI has been found not to be superior to WC as an indicator of hepatic steatosis and steatohepatitis in a recent publication [16].

The fact that BMI, a marker of global adiposity, is also a reliable marker of visceral adiposity is easily explained by the fact that visceral adipose tissue increases in a quasi-linear manner with BMI in both sexes [37]. Finally, only the WHR showed statistically significant correlations with sex steroids, a not so surprising finding considering that, among the indexes studied here, only the WHR takes into account upper and lower body fat distribution.

In conclusion, in young men and women presenting with wide ranges of weight, visceral adiposity and insulin resistance, WC and BMI are not only the simplest to obtain, but are also the most accurate surrogate markers of visceral adiposity, good indicators or insulin resistance, and powerful predictors of the presence of hepatic steatosis with fairly good sensitivity and specificity.

## References

[1] Bjorntorp P (1993) Visceral obesity: a “civilization syndrome”. Obes Res 1:206–222. 16350574 10.1002/j.1550-8528.1993.tb00614.x

[2] Kissebah AH (1991) Insulin resistance in visceral obesity. Int J Obes 15 Suppl 2109–115. 1794931

[3] Fernandez-Real JM, Ricart W (2003) Insulin resistance and chronic cardiovascular inflammatory syndrome. Endocr Rev 24:278–301. 12788800 10.1210/er.2002-0010

[4] Lemieux S, Despres JP (1994) Metabolic complications of visceral obesity: contribution to the aetiology of type 2 diabetes and implications for prevention and treatment. Diabete Metab 20:375–393. 7843469

[5] Chan DC, Barrett HP, Watts GF (2004) Dyslipidemia in visceral obesity: mechanisms, implications, and therapy. Am J Cardiovasc Drugs 4:227–246. 15285698 10.2165/00129784-200404040-00004

[6] Escobar-Morreale HF, San Millan JL (2007) Abdominal adiposity and the polycystic ovary syndrome. Trends Endocrinol Metab 18:266–272. 17693095 10.1016/j.tem.2007.07.003

[7] Corona G, Rastrelli G, Morelli A, Vignozzi L, Mannucci E, et al (2011) Hypogonadism and metabolic syndrome. J Endocrinol Invest 34:557–567. 21720206 10.3275/7806

[8] Luyckx FH, Lefebvre PJ, Scheen AJ (2000) Non-alcoholic steatohepatitis: association with obesity and insulin resistance, and influence of weight loss. Diabetes Metab 26:98–106. 10804323

[9] Mathieu P, Poirier P, Pibarot P, Lemieux I, Despres JP (2009) Visceral obesity: the link among inflammation, hypertension, and cardiovascular disease. Hypertension 53:577–584. 19237685 10.1161/HYPERTENSIONAHA.108.110320

[10] Giovannucci E, Michaud D (2007) The role of obesity and related metabolic disturbances in cancers of the colon, prostate, and pancreas. Gastroenterology 132:2208–2225. 17498513 10.1053/j.gastro.2007.03.050

[11] Lee M, Aronne LJ (2007) Weight management for type 2 diabetes mellitus: global cardiovascular risk reduction. Am J Cardiol 99:68B–79B. 17307059 10.1016/j.amjcard.2006.11.007

[12] Shuster A, Patlas M, Pinthus JH, Mourtzakis M (2012) The clinical importance of visceral adiposity: a critical review of methods for visceral adipose tissue analysis. Br J Radiol 85:1–10. 21937614 10.1259/bjr/38447238 PMC3473928

[13] Alberti KG, Zimmet P, Shaw J (2005) The metabolic syndrome–a new worldwide definition. Lancet 366:1059–1062. 16182882 10.1016/S0140-6736(05)67402-8

[14] Amato MC, Giordano C, Galia M, Criscimanna A, Vitabile S, et al (2010) Visceral Adiposity Index: a reliable indicator of visceral fat function associated with cardiometabolic risk. Diabetes Care 33:920–922. 20067971 10.2337/dc09-1825 PMC2845052

[15] Petta S, Amato MC, Di Marco V, Camma C, Pizzolanti G, et al (2012) Visceral adiposity index is associated with significant fibrosis in patients with non-alcoholic fatty liver disease. Aliment Pharmacol Ther 35:238–247. 22117531 10.1111/j.1365-2036.2011.04929.x

[16] Vongsuvanh R, George J, McLeod D, van der Poorten D (2012) Visceral adiposity index is not a predictor of liver histology in patients with non-alcoholic fatty liver disease. J Hepatol 57:392–398. 22521350 10.1016/j.jhep.2012.03.013

[17] Mohammadreza B, Farzad H, Davoud K, Fereidoun Prof AF (2012) Prognostic significance of the complex “Visceral Adiposity Index” vs. simple anthropometric measures: Tehran lipid and glucose study. Cardiovasc Diabetol 11:20. 22394430 10.1186/1475-2840-11-20 PMC3376032

[18] Al-Daghri NM, Al-Attas OS, Alokail MS, Alkharfy KM, Charalampidis P, et al (2013) Visceral adiposity index is highly associated with adiponectin values and glycaemic disturbances. Eur J Clin Invest 43:183–189. 23278387 10.1111/eci.12030

[19] Al-Daghri NM, Al-Attas OS, Alokail M, Alkharfy K, Wani K, et al. (2013) Does Visceral Adiposity Index signify early metabolic risk in children and adolescents? Association with insulin resistance, adipokines and subclinical inflammation. Pediatr Res. 10.1038/pr.2013.229 24296798

[20] Elisha B, Messier V, Karelis A, Coderre L, Bernard S, et al (2013) The Visceral Adiposity Index: Relationship with cardiometabolic risk factors in obese and overweight postmenopausal women - A MONET group study. Appl Physiol Nutr Metab 38:892–899. 23855278 10.1139/apnm-2012-0307

[21] Mazzuca E, Battaglia S, Marrone O, Marotta AM, Castrogiovanni A, et al**.** (2013) Gender-specific anthropometric markers of adiposity, metabolic syndrome and visceral adiposity index (VAI) in patients with obstructive sleep apnea. J Sleep Res. 10.1111/jsr.12088 24118617

[22] Du T, Sun X, Huo R, Yu X (2013) Visceral adiposity index, hypertriglyceridemic waist and risk of diabetes: the China Health and Nutrition Survey 2009. Int J Obes. 10.1038/ijo.2013.181 24048141

[23] Borruel S, Fernandez-Duran E, Alpanes M, Marti D, Alvarez-Blasco F, et al (2013) Global adiposity and thickness of intraperitoneal and mesenteric adipose tissue depots are increased in women with polycystic ovary syndrome (PCOS). J Clin Endocrinol Metab 98:1254–1263. 23386652 10.1210/jc.2012-3698

[24] World Health Organization (2000) Obesity: preventing and managing the global epidemic. Report of a WHO Consultation. In: World Health Organization Monograph Series , editor. WHO Technical Report Series 894. Geneva: World Health Organization. 11234459

[25] Zawadzki JK, Dunaif A (1992) Diagnostic criteria for polycystic ovary syndrome: Towards a rational approach. In: Dunaif A, Givens JR, Haseltine FP, Merriam GReditors. Polycystic ovary syndrome. Boston: Blackwell Scientific Publications. pp. 377–384.

[26] The Rotterdam ESHRE/ASRM-sponsored PCOS consensus workshop group (2004) Revised 2003 consensus on diagnostic criteria and long-term health risks related to polycystic ovary syndrome (PCOS). Hum Reprod 19:41–47. 14688154 10.1093/humrep/deh098

[27] Azziz R, Carmina E, Dewailly D, Diamanti-Kandarakis E, Escobar-Morreale HF, et al (2006) Position statement: criteria for defining polycystic ovary syndrome as a predominantly hyperandrogenic syndrome: an Androgen Excess Society guideline. J Clin Endocrinol Metab 91:4237–4245. 16940456 10.1210/jc.2006-0178

[28] Escobar-Morreale HF, Lasuncion MA, Sancho J (2000) Treatment of hirsutism with ethinyl estradiol-desogestrel contraceptive pills has beneficial effects on the lipid profile and improves insulin sensitivity. Fertil Steril 74:816–819. 11020530 10.1016/s0015-0282(00)00718-4

[29] Escobar-Morreale HF, Sanchon R, San Millan JL (2008) A prospective study of the prevalence of nonclassical congenital adrenal hyperplasia among women presenting with hyperandrogenic symptoms and signs. J Clin Endocrinol Metab 93:527–533. 18000084 10.1210/jc.2007-2053

[30] Matsuda M, DeFronzo RA (1999) Insulin sensitivity indices obtained from oral glucose tolerance testing: comparison with the euglycemic insulin clamp. Diabetes Care 22:1462–1470. 10480510 10.2337/diacare.22.9.1462

[31] Stolk RP, Wink O, Zelissen PM, Meijer R, van Gils AP, et al (2001) Validity and reproducibility of ultrasonography for the measurement of intra-abdominal adipose tissue. Int J Obes Relat Metab Disord 25:1346–1351. 11571598 10.1038/sj.ijo.0801734

[32] Kim SK, Kim HJ, Hur KY, Choi SH, Ahn CW, et al (2004) Visceral fat thickness measured by ultrasonography can estimate not only visceral obesity but also risks of cardiovascular and metabolic diseases. The American Journal of Clinical Nutrition 79:593–599. 15051602 10.1093/ajcn/79.4.593

[33] Ribeiro-Filho FF, Faria AN, Kohlmann O Jr, Ajzen S, Ribeiro AB, et al (2001) Ultrasonography for the Evaluation of Visceral Fat and Cardiovascular Risk. Hypertension 38:713–717. 11566963 10.1161/01.hyp.38.3.713

[34] Liu KH, Chan YL, Chan WB, Kong WL, Kong MO, et al (2003) Sonographic measurement of mesenteric fat thickness is a good correlate with cardiovascular risk factors: comparison with subcutaneous and preperitoneal fat thickness, magnetic resonance imaging and anthropometric indexes. Int J Obes Relat Metab Disord 27:1267–1273. 14513076 10.1038/sj.ijo.0802398

[35] Ahn SG, Lim HS, Joe DY, Kang SJ, Choi BJ, et al (2008) Relationship of epicardial adipose tissue by echocardiography to coronary artery disease. Heart 94:e7. 17923467 10.1136/hrt.2007.118471

[36] Saadeh S, Younossi ZM, Remer EM, Gramlich T, Ong JP, et al (2002) The utility of radiological imaging in nonalcoholic fatty liver disease. Gastroenterology 123:745–750. 12198701 10.1053/gast.2002.35354

[37] Ferrannini E, Sironi AM, Iozzo P, Gastaldelli A (2008) Intra-abdominal adiposity, abdominal obesity, and cardiometabolic risk. Eur Heart J Suppl 10 B4–B10.

